# Integrating Patient-Reported Outcomes into Clinical Pathways in Atrial Fibrillation: A Framework Aligned with the AF-CARE Model

**DOI:** 10.3390/diagnostics16091398

**Published:** 2026-05-06

**Authors:** Emma Sokolova, Olav Goetz, Ketija Grīnberga, Sevinc Elif Sen, Kaspars Kupics, Aija Mača-Kalēja, Ainārs Rudzītis, Daiga Behmane, Oskars Kalējs

**Affiliations:** 1 Department of Internal Diseases, Riga Stradiņš University, LV-1007 Riga, Latvia; 2 Doctoral Study Programme in Medicine, Rīga Stradiņš University, LV-1007 Riga, Latvia; 3 Centre of Clinical Diagnostics, Riga East Clinical University Hospital, LV-1007 Riga, Latvia; 4 Faculty of Health Economics, APOLLON University of Applied Sciences, 28195 Bremen, Germany; 5 Independent Researcher, Dublin, Ireland; 6 Latvian Centre of Cardiology, Pauls Stradiņš Clinical University Hospital, LV-1002 Riga, Latvia; 7 Faculty of Public Health and Social Welfare, Rīga Stradiņš University, LV-1007 Riga, Latvia

**Keywords:** atrial fibrillation, quality of life, patient-reported outcomes, health policy, AF-CARE pathway, value-based healthcare, healthcare systems, PROM integration, clinical pathways, health system integration

## Abstract

**Background**: Atrial fibrillation (AF) significantly impacts the clinical and financial burden of healthcare systems worldwide. Patient-reported outcomes (PROMs) are considered a core element of patient-centered care. However, the integration of PROMs into AF clinical pathways remains limited. **Objectives**: In this study, we aimed to develop a policy-oriented framework for integrating PROMs into AF management, building on QoL assessment and the AF-CARE pathway. Methods: We compared the selected instruments (AFEQT, SF-36, EQ-5D, and AFSS) to examine how different QoL domains are represented. We then considered how the missing domains relate to routine care and healthcare delivery. On this basis, we constructed a conceptual framework linking PROM domains with clinical and policy decisions, as well as with diagnostic and follow-up strategies in AF. **Results**: Existing PROMs capture symptom burden reasonably well, but they do not fully reflect several domains that shape the daily experience of patients with AF, including cognition, sleep quality, sexual health, and economic burden. The proposed framework places PROMs within the AF-CARE components and illustrates how they can be used in routine practice—not only for long-term monitoring, but also to improve clinical decision-making, and better align with value-based healthcare strategies at both the clinical and system levels. It also outlines a practical approach to incorporating PROMs into everyday care and into broader health-system decision-making. **Conclusions**: Integrating PROMs into AF-CARE requires a system-level redesign rather than isolated tool implementation. Within the proposed framework, the question is no longer whether PROMs should be used in AF care, but how to integrate them in a way that meaningfully influences clinical decisions and patient outcomes.

## 1. Introduction

Atrial fibrillation (AF) is the most common sustained cardiac arrhythmia worldwide, affecting an estimated 37–40 million individuals [1,2]. It is associated with increased risk of stroke, heart failure, hospitalization, and mortality, placing a substantial clinical and economic burden on healthcare systems [3,4]. At the same time, AF has an important impact on patients’ quality of life (QoL), influencing physical functioning, emotional well-being, social participation, and overall health perception [5]. Symptom burden in AF is often weakly correlated with traditional clinical setting, highlighting the limitations of conventional outcome assessment [6]. For this reason, QoL has become an increasingly important endpoint in both clinical research and routine care.

Patient-reported outcome measures (PROMs) aim to capture aspects of disease burden that are not reflected in physiological tests or imaging findings. In practice, they provide insights into dimensions of the patient experience that remain largely invisible to standard clinical tools [7]. PROMs are now explicitly recommended in recent clinical guidelines, including the latest European Society of Cardiology (ESC) guidelines [8], which emphasize patient-centered care through structured pathways such as AF-CARE [3]. Despite this, implementation of PROMs in clinical practice remains limited [9,10,11,12,13]. In many settings, AF care still prioritizes outcomes that are straightforward to measure, while domains that are more relevant to patients receive less systematic attention. Available QoL instruments—including disease-specific tools such as the Atrial Fibrillation Effect on Quality-of-Life (AFEQT) questionnaire and generic instruments such as EQ-5D and SF-36—vary considerably in their domain coverage, sensitivity, and clinical applicability [14,15,16]. Although AFEQT is widely regarded as one of the most comprehensive AF-specific instruments, it does not fully capture several domains that are increasingly recognized as clinically relevant, including cognitive function, sleep quality, sexual health, and economic burden [17]. This limitation points to a broader gap between how outcomes are measured and how patients experience the disease. For example, studies suggest that up to 30–40% of patients with AF report cognitive complaints or sleep disturbances, both of which are associated with reduced quality of life and increased healthcare utilization [6,10,18,19].

In most healthcare systems, PROMs are still used primarily as research contexts rather than as an integral part of routine clinical decision-making or policy planning [9]. As a result, their potential to inform treatment strategies, guide resource allocation, and improve health system performance remains underused. The shift toward value-based healthcare (VBHC) [10,18,20] further highlights the importance of integrating patient-centered outcomes [19,21] into structured care pathways [22,23]. VBHC frameworks prioritize outcomes that matter to patients relative to the cost of delivering care, which requires more comprehensive and multidimensional approaches to outcome measurement [22]. In AF, where hospitalizations account for a substantial proportion of healthcare expenditure, incorporating PROMs into routine care has the potential to improve both clinical outcomes and system efficiency [4,24].

This study builds upon existing evidence by moving beyond the comparison of QoL instruments and proposing a policy-oriented framework for integrating PROMs into AF management. Current AF care prioritizes clinically measurable outcomes yet does not consistently capture domains that are highly relevant to patients, including cognition, sleep quality, sexual health, and economic burden. This misalignment may, in part, limit the real-world effectiveness of AF management [6]. By aligning QoL domains with the AF-CARE pathway and linking patient-reported outcomes to clinical and health system decision points, the proposed framework aims to support patient-centered, value-based care in atrial fibrillation. In this context, PROMs may function as complementary diagnostic tools for symptom assessment and clinical decision-making, enabling more precise characterization of symptom burden and disease impact beyond conventional clinical parameters [13,22]. Accordingly, this study argues that patient-reported outcomes should be repositioned as central drivers of clinical decision-making and proposes a policy-oriented framework for integrating PROMs into AF care based on the AF-CARE model.

This paper (1) reviews existing PROM instruments, (2) identifies critical gaps, and (3) proposes an implementation framework aligned with the AF-CARE pathway.

## 2. Materials and Methods

This study is a conceptually driven analytical review aimed at developing a policy-oriented framework for integrating patient-reported outcomes into atrial fibrillation management within the AF-CARE model. It does not seek to provide a systematic or exhaustive synthesis of the literature, but rather to identify key gaps in existing quality-of-life instruments and to translate these findings into a structured, clinically applicable framework.

The analysis focused on evaluating commonly used QoL instruments in AF and identifying domains that are insufficiently represented in current assessments.

A literature search was conducted using PubMed, Scopus, Web of Science, and the Cochrane Library, covering publications from database inception through December 2024. Search terms included combinations of: “atrial fibrillation”, “quality of life”, “patient-reported outcomes”, “PROMs”, “AFEQT”, “EQ-5D”, “SF-36”, “MLHFQ”, “AFSS” [25,26]. A summary of the psychometric properties and scoring criteria of each instrument is provided in Table S1. Medical Subject Headings (MeSH) and Boolean operators were used to refine the search strategy. Reference lists of included studies were also screened to identify additional relevant publications. Studies were selected based on their relevance to QoL assessment in AF populations, with priority given to peer-reviewed publications evaluating or applying validated PROM instruments in adult patients with AF. A total of 65 studies published between 1992 and 2024 informed the analysis, comprising randomised controlled trials, observational cohort studies, and registry-based analyses. Exclusion criteria: case reports; editorials and opinion pieces; studies without AF-specific QoL outcomes; non-English publications.

QoL measures were evaluated in detail by focusing on five instruments that are most commonly applied, have been validated in AF populations, and demonstrate clear clinical relevance: AFEQT (Atrial Fibrillation Effect on Quality-of-Life) [14]; EQ-5D (EuroQoL 5-Dimensions) [15]; SF-36 (Short Form Health Survey) [16]; MLHFQ (Minnesota Living with Heart Failure Questionnaire) [27]; AFSS (Atrial Fibrillation Severity Scale) [28].

A structured domain framework was developed to assess the comprehensiveness of each instrument. Ten QoL domains were selected based on literature, clinical relevance, and international outcome measurement standards [29]:

1. Physical functionality
2. Symptoms
3. Emotional well-being
4. Social functionality
5. Cognitive function
6. Treatment satisfaction
7. Economic burden
8. Health perception
9. Sexual health
10. Sleep quality

Each instrument was evaluated according to the degree to which it covered each domain, categorised as full, partial or none. Full coverage was defined as the presence of one or more dedicated items or subscales directly measuring the target domain. Partial coverage referred to limited or indirect assessment through items embedded within broader constructs. None indicated that the domain was not addressed by the instrument. The findings were combined in a comparative domain matrix. Subsequently, a gap analysis was conducted to highlight underrepresented domains and assess their importance from both clinical and policy perspectives.

Based on the gap analysis, a conceptual framework was developed to integrate QoL domains into the AF-CARE pathway and broader healthcare system structures.

The framework was constructed using three levels:

• Core QoL domains (captured by existing PROMs)
• Extended domains (underrepresented but clinically and socially relevant)
• System integration layer (linking PROMs to clinical decision-making and policy outcomes)

To enhance applicability at the health system level, identified domains were mapped to AF-CARE pathway components, clinical decision points, and healthcare system outcomes (hospitalizations, costs, and resource utilization). This approach enabled the translation of PROM data into actionable insights for both clinical practice and health policy planning.

**Figure 1:**
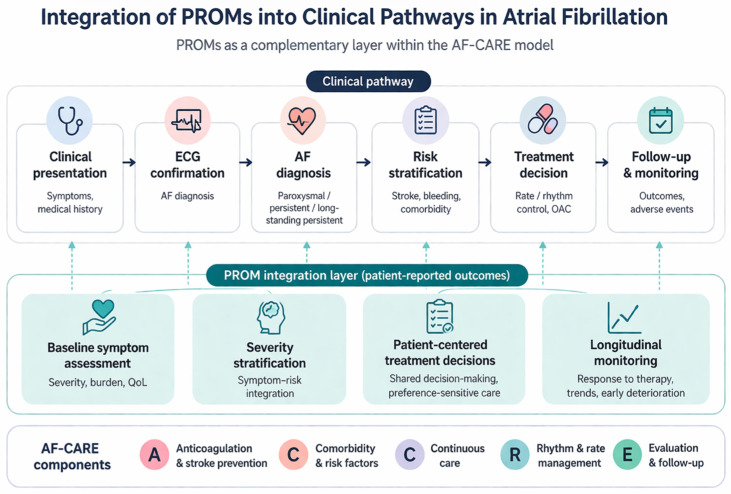
Integration of PROMs into clinical pathways. ECG = electrocardiogram.

During the preparation of this manuscript, the authors used the assistance of ChatGPT for the purposes for visual output of Figure 1. The authors have reviewed and edited the output and take full responsibility for the content of this publication.

## 3. Results

The literature search identified a broad range of studies evaluating QoL in patients with AF. Following screening and eligibility assessment (see Methods), a total of 65 studies published between 1992 and 2024 were included in the final analysis. The included studies comprised randomized controlled trials, observational cohort studies, and registry-based analyses. Across these studies, five QoL instruments were consistently identified as the most frequently used and validated in AF populations: AFEQT, EQ-5D, SF-36, MLHFQ, and AFSS.

### 3.1. Comparative Analysis of QoL Instruments

The analysis revealed substantial variability in domain coverage across instruments, with important implications for both clinical practice and health policy.

Table 1 summarizes the structural characteristics and domain coverage of commonly used PROM instruments in AF [18,23].

Table 2 summarizes the clinical relevance of key patient-reported outcome domains in atrial fibrillation and their alignment with the ESC 2024 AF-CARE framework [8]. The integration of PROM domains [11,13] into AF-CARE components demonstrates how patient-reported data can support clinical assessment, clinical decision-making, and continuous evaluation within structured care pathways.

#### 3.1.1. QoL Instruments, Domain Coverage and Variability

Among the AF-specific instruments, AFEQT emerged as the most comprehensive. It covers key domains such as symptoms, physical functioning, emotional well-being, treatment satisfaction, and overall health perception. At the same time, its coverage is less consistent in areas such as cognition and economic burden, and domains like sexual health and sleep quality are not systematically assessed.

In contrast, EQ-5D provides a much more limited perspective. It focuses mainly on general health status, mobility, and emotional well-being. Although it is widely used in economic evaluations and allows comparisons across diseases, it is less sensitive to AF-specific symptom burden and functional limitations.

SF-36 offers a broader multidimensional assessment than EQ-5D, with coverage of physical, emotional, and social domains. However, this broader scope comes at the expense of disease specificity, as AF-related symptoms and treatment-related burden are not explicitly captured.

The Minnesota Living with Heart Failure Questionnaire (MLHFQ), although originally developed for heart failure, shows some applicability in AF populations, likely due to overlapping symptom profiles. It captures certain domains, including aspects of cognition and sleep, but does not provide AF-specific detail.

The Atrial Fibrillation Symptom Severity Scale (AFSS), by contrast, is focused primarily on symptom severity and frequency. While this allows for a more detailed characterization of arrhythmia-related symptoms, it does not extend to broader quality-of-life domains.

Across all instruments, several domains were consistently represented, particularly physical functioning, emotional well-being, and general health perception. Other domains were much less visible. Cognitive function, sleep quality, sexual health, and economic burden were either only partially covered or not addressed at all. Notably, sexual health was absent across all five instruments, while economic burden and cognitive function were only inconsistently represented.

Taken together, these findings suggest that no single instrument currently provides a comprehensive representation of the multidimensional burden experienced by patients with AF.

#### 3.1.2. Identification of Gaps in QoL Measurement

The gap analysis suggests a clear mismatch between currently available QoL instruments and the multidimensional burden experienced by patients with AF.

Several domains appear to be consistently underrepresented. Cognitive function is one of them, despite growing evidence linking AF with cognitive decline and an increased risk of dementia. Sleep quality represents another important domain, particularly given its association with AF triggers and recurrence. Sexual health, although closely related to overall well-being, is rarely assessed in cardiovascular research. Economic burden—including both direct and indirect costs—also remains insufficiently captured, despite its clear relevance for patients and health systems. Taken together, these observations point to a broader limitation of existing PROMs. Domains that are highly relevant for patients, and potentially important for clinical decision-making, are not systematically incorporated into routine assessment. As a result, some aspects of disease burden remain effectively “invisible” within clinical pathways.

This gap may have practical consequences. When relevant domains are not measured, they are less likely to be addressed in clinical care, which can limit both treatment effectiveness and efficient use of healthcare resources [9,13]. In this sense, incomplete measurement may translate into incomplete management. Consistent with this, several domains remain only partially represented across available instruments, which may contribute to gaps in clinical assessment and, in some cases, suboptimal treatment decisions (Table 3). In addition to the domains outlined above, social functioning was also found to be inconsistently captured across PROM instruments.

While Table 1 provides an overview of the structural characteristics and domain coverage of PROM instruments, Table 3 focuses specifically on identifying gaps in domain representation and their potential clinical implications.

Table 3 highlights key gaps in PROM-based assessment and their potential clinical implications within the AF-CARE pathway. Several domains relevant to disease burden and prognosis such as cognitive function, sleep quality, and economic burden are not consistently captured, potentially limiting clinical accuracy and the completeness of clinical assessment and the comprehensiveness of patient evaluation.

Several domains that are relevant for both disease burden and prognosis—such as cognitive function, sleep quality, and economic burden—are not consistently captured. This may limit the completeness of patient evaluation and, in some cases, reduce clinical accuracy.

The analysis demonstrated that QoL domains are not only patient-centered outcomes but are also closely linked to clinical and system-level indicators. Symptom burden was associated with treatment response and rhythm control success. Emotional and social domains were linked to treatment adherence and healthcare utilization. Sleep disturbances and cognitive impairment were associated with increased risk of recurrence and complications. Economic burden was directly related to hospitalization rates and healthcare costs. These relationships indicate that QoL assessment has the potential to inform both clinical decision-making and resource allocation. This structured linkage between PROM gaps and clinical implications represents a key contribution of the present analysis.

### 3.2. Framework Development

Based on the identified gaps and the observed interactions between domains, we developed a policy-oriented QoL framework for AF (Figure 1). The framework is organized as a structured care pathway rather than a static set of domains. It begins with initial clinical presentation and confirmation of AF, followed by the integration of PROMs into severity stratification and combined clinical–PROM assessment. These steps inform personalized treatment strategies and support a more integrated, patient-centered approach to management. Within this pathway, core domains—such as symptoms, physical functioning, and emotional well-being—are complemented by extended domains, including cognition, sleep quality, sexual health, and economic burden, which provide additional context for clinical decision-making. In this way, QoL assessment is not treated as a separate layer, but as an integral component of the clinical and management process.

Figure 1 demonstrates the role of patient-reported outcome measures (PROMs) as a complementary layer within AF care. Following clinical presentation and electrocardiographic (ECG) confirmation of AF, PROMs are integrated into subsequent stages of care, including symptom assessment, severity stratification, treatment decision-making, and follow-up. PROMs are not used for the diagnosis of AF, but rather provide additional patient-centered information that supports clinical evaluation, decision-making and longitudinal management within the AF-CARE framework.

#### Mapping to AF-CARE Pathway

The framework demonstrates alignment between QoL domains and the AF-CARE pathway:

• C (Comorbidity and risk factors) → cognition, sleep
• A (Avoid stroke) → health perception and functional status
• R (Rate and rhythm control) → symptom burden
• E (Evaluation and follow-up) → dynamic PROM monitoring

This mapping highlights the role of PROMs as continuous feedback mechanisms within clinical pathways.

### 3.3. Implications for Health Systems

The integration of QoL domains into structured care pathways allows PROMs to function beyond descriptive tools, enabling early identification of high-risk patients, improved targeting of interventions, reduction in hospitalizations, and more efficient allocation of healthcare resources. These findings support the use of PROM-based frameworks as instruments for both clinical optimization and health policy development.

#### 3.3.1. Policy Implications and System Integration

The integration of PROMs into AF management requires a shift from measurement-focused approaches to system-level implementation strategies. Current clinical practice often treats PROMs as supplementary tools rather than core decision-making instruments.

From a health policy perspective, three critical challenges emerge: (1) fragmentation of care pathways—PROMs are not systematically embedded within clinical workflows, limiting their impact on treatment decisions; (2) lack of standardized domains across instruments—the absence of consensus on core QoL domains hinders comparability and scalability at the system level; and (3) insufficient linkage to health system outcomes—PROM data are rarely connected to resource utilization, hospitalization rates, or cost-effectiveness analyses. To address these challenges, the proposed framework introduces a structured integration of PROMs into the AF-CARE pathway. This allows patient-reported outcomes to function as dynamic triggers for clinical reassessment, treatment adjustment, and resource allocation.

To further operationalize the integration of PROMs within the AF-CARE pathway, specific PROM-based actions can be defined for each component:

• C (Comorbidity and risk factors): PROM domains such as cognitive function and sleep quality may be used to identify comorbid conditions and risk modifiers. For example, reported cognitive decline may prompt neurological evaluation, while impaired sleep quality may trigger screening for sleep apnea or other modifiable risk factors.
• A (Avoid stroke): Although PROMs do not directly assess thromboembolic risk, domains such as health perception and functional status may provide additional context for treatment adherence and patient engagement, potentially influencing anticoagulation strategies.
• R (Rate and rhythm control): Symptom-related PROM domains may support decisions between rate and rhythm control strategies, help evaluate treatment response, and guide therapy adjustments based on patient-reported burden.
• E (Evaluation and follow-up): Longitudinal PROM monitoring enables tracking of symptom progression, treatment effectiveness, and quality-of-life changes over time. Changes in PROM scores may act as triggers for clinical reassessment and modification of treatment strategies.

This structured linkage between PROM domains and clinical actions represents an operational extension of the AF-CARE pathway, translating patient-reported data into actionable clinical insights.

#### 3.3.2. PROM Integration

Integrating PROMs into AF care requires a structured approach at several levels (Table 4).

At the clinical level, PROMs can be incorporated into routine workflows, for example, at baseline and during follow-up visits (e.g., every 3–6 months). When used consistently, they may help to contextualize treatment response, support decisions on rhythm versus rate control, and prompt reassessment when patient-reported status deteriorates.

At the organizational level, PROM data need to be integrated into electronic health records and interpreted within a multidisciplinary context. In practice, this often involves collaboration between cardiologists and primary care providers, which may improve continuity of care across settings.

At the policy level, PROMs could be linked to performance indicators, reimbursement models, and national registries. This would allow patient-reported outcomes to contribute to health system evaluation, resource allocation, and quality benchmarking, although such approaches are still developing in many settings.

Taken together, this multilevel approach emphasizes that PROMs are most useful when they are not only collected, but also actively used. Linking patient-reported outcomes with clinical workflows and policy processes may help to narrow the gap between patient experience and system-level performance.

**Figure 2:**
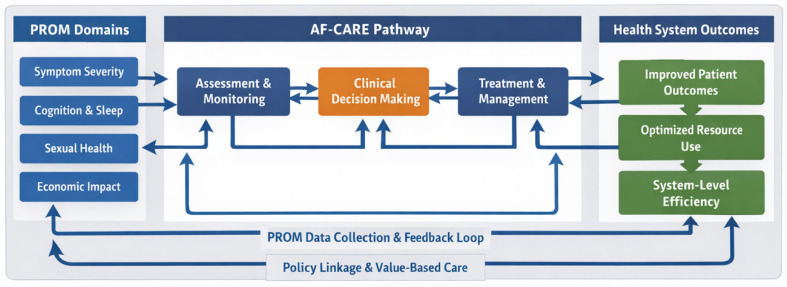
Policy-integrated framework for quality-of-life assessment in AF.

The framework emphasizes the transition from outcome measurement to outcome-driven care (Figure 2).

The figure illustrates the PROM-driven AF care cycle—a continuous feedback loop in which patient-reported outcomes function not as passive measurements but as active drivers of clinical decision-making and healthcare system adaptation. It depicts the interactions between PROM domains, clinical decision-making, policy-level planning within the AF-CARE pathway, and health system outcomes. Bidirectional arrows indicate the dynamic relationships between clinical decisions, health system outcomes, and continuous feedback loops within the AF-CARE pathway, reflecting the integration of PROMs into value-based healthcare.

### 3.4. Health System Context: The Case of Latvia

Atrial fibrillation represents a growing challenge for healthcare systems across Europe, including Latvia, where population ageing and the increasing prevalence of cardiovascular diseases contribute to a rising AF burden. Although precise national epidemiological data remain limited, European estimates suggest that AF affects approximately 2–4% of the adult population, with substantially higher prevalence in older age groups [4,30].

In Latvia, AF-related hospitalizations constitute a significant proportion of cardiovascular healthcare utilization, reflecting both the clinical burden of the disease and the episodic nature of care delivery. Care for patients with AF is provided through a combination of specialized cardiology services and primary care. A recent study at Pauls Stradiņš Clinical University Hospital evaluated quality of life using SF-36 in Latvian patients undergoing AF ablation, providing initial evidence on QoL assessment in this population [31]. However, the integration of patient-reported outcomes into routine clinical pathways remains limited. Clinical decision-making is largely based on objective parameters, while patient-reported dimensions such as quality of life are not consistently assessed or integrated into treatment decisions. As in other Central and Eastern European countries, the healthcare system faces challenges related to resource allocation, care coordination, and integration between primary and specialist services.

At present, the use of patient-reported outcome measures in Latvia remains limited and is not systematically embedded within routine clinical workflows [32]. Clinical decision-making is largely based on objective parameters, while patient-reported dimensions—such as quality of life—are not consistently assessed or integrated into treatment decisions.

From a system-level perspective, several barriers to PROM implementation can be identified [19,33,34]. These include the absence of standardized integration within electronic health records, limited experience with routine PROM collection, and a lack of linkage between patient-reported outcomes and healthcare performance indicators or reimbursement mechanisms [35].

In this context, frameworks aligned with international models such as the AF-CARE pathway may provide a structured approach for integrating PROMs into routine care. Such approaches could support improved identification of high-risk patients, enhance coordination between levels of care, and contribute to more efficient use of healthcare resources.

Although the implementation of PROM-based strategies in Latvia remains at an early stage, the increasing emphasis on value-based healthcare creates an opportunity to incorporate patient-centered outcomes into national care models and health policy planning [24,32]. Future research should focus on generating Latvia-specific data on AF prevalence, healthcare utilization, and the feasibility of PROM integration in routine practice.

## 4. Discussion

Despite growing recognition of patient-reported outcomes in AF, routine clinical practice continues to rely mainly on traditional endpoints such as rhythm status, hospitalization, and mortality. These metrics remain important, but they do not fully reflect how patients experience the disease. As a result, a gap persists between what is measured in clinical settings and what patients perceive as meaningful improvement. Instruments such as AFEQT have helped to address this issue to some extent. However, their use in routine care is still inconsistent and often limited to descriptive assessment rather than active decision support.

In this context, the present study focuses less on comparing PROM instruments and more on how they can be integrated into clinical pathways and health system design. Although PROMs are increasingly discussed, they are still frequently treated as secondary outcomes rather than as inputs that shape clinical decisions [13,17]. This limits their influence on treatment strategies, particularly in areas such as rhythm versus rate control.

A central observation of this analysis is that most PROMs—including AFEQT, EQ-5D, and SF-36—were developed primarily for measurement. They provide useful descriptive information but are rarely used as actionable tools within clinical workflows. This reflects a broader issue in healthcare, where outcome measurement is often not directly linked to care delivery [13,17,22]. In practice, PROMs remain more common in research settings than in routine clinical or policy use [18,23].

The framework proposed here attempts to address this gap by positioning PROMs as part of the clinical process rather than as separate endpoints. Within this approach, QoL measures can function as signals that prompt reassessment, support treatment adjustments, and contribute to longitudinal monitoring.

While individual gaps in PROM domain coverage have been previously reported, the present study extends existing literature by systematically linking these gaps to their potential clinical consequences and integrating them within the AF-CARE pathway.

An important aspect of this framework is the inclusion of domains that are currently underrepresented in most instruments, such as cognition, sleep quality, sexual health, and economic burden. These domains are increasingly recognized as clinically relevant. For example, cognitive impairment in AF has been linked to microembolism and chronic cerebral hypoperfusion, while sleep disturbances may both trigger and result from AF episodes [6,15]. Similarly, the economic burden of AF—largely driven by hospitalizations—remains a key concern at the system level. By incorporating these domains, the framework creates a link between patient-reported experience and system-level outcomes, including hospitalizations, costs, and care coordination [36]. This may help to align patient-centered care with broader healthcare priorities.

The findings are broadly consistent with recent guideline developments, particularly the 2024 ESC AF guidelines, which emphasize patient-centered care within the AF-CARE pathway. The framework presented here can be seen as one way of operationalizing these principles by embedding PROMs within different stages of clinical decision-making. At the same time, several challenges need to be acknowledged. Integrating PROMs into routine care may increase workload, contribute to measurement fatigue, and introduce data that are not always straightforward to interpret. These concerns are especially relevant in busy clinical environments. However, their impact depends largely on how PROMs are implemented. Digital data collection, short-form instruments, and selective use at key decision points may reduce the burden while maintaining clinical usefulness. In other areas of medicine, PROM integration has been associated with improved symptom control, patient satisfaction, and, in some settings, survival [18]. Whether similar effects can be achieved in AF remains to be confirmed.

The relevance of this approach may be particularly evident in healthcare systems such as those in Latvia, where AF prevalence is increasing, and resources are limited. Current models of care remain largely clinician-driven, with limited incorporation of patient-reported data. Introducing PROMs into routine practice could support better coordination between primary and specialist care, improve identification of high-risk patients, and contribute to more efficient use of resources.

From a practical perspective, PROMs may be most useful when collected at baseline and during follow-up, for example, at 3–6-month intervals. Changes in AFEQT scores could help to contextualize treatment response, while PROM trajectories may support decisions regarding rhythm control strategies. Integration into digital platforms will likely be essential for scalability [37].

Overall, the challenge is not the absence of PROM instruments, but how they are used. Moving from measurement to meaningful integration will require changes at the level of clinical workflows, digital infrastructure, and health policy.

This study does not include primary empirical data and should be interpreted as a conceptual framework requiring validation.

### 4.1. Strengths and Limitations

This study has several notable strengths. First, to our knowledge, this is the first framework that systematically maps PROM domain gaps to AF-CARE decision points with specific clinical actions proposed at each stage. Second, the framework is grounded in a review of 65 studies and aligned with the 2024 ESC AF-CARE guidelines. Third, the inclusion of both clinical and policy relevance for each domain bridges the gap between patient-centered measurement and health system planning. Fourth, the multilevel implementation framework (Table 4) provides actionable indicators at clinical, organizational, and policy levels. Fifth, the framework has been operationalized into an interactive decision-support tool, which is described in a companion study [38].

Several limitations should also be considered. The present study is based on a conceptual synthesis of existing evidence and does not include primary patient-level data. Although the framework is grounded in published literature and current guidelines, its practical value will depend on validation in real-world settings. Future research should therefore examine its impact on clinical outcomes, healthcare utilization, and cost-effectiveness across different healthcare systems.

### 4.2. Potential Harms and Implementation Barriers

While the proposed framework aims to improve patient-centered AF care, several implementation barriers and potential unintended consequences should be acknowledged.

First, routine PROM collection may increase clinician workload, particularly in busy outpatient settings where consultation time is already limited [13]. If PROM data are collected but not meaningfully integrated into clinical workflows, the exercise risks becoming an administrative burden without corresponding clinical benefit—a phenomenon described as “measurement fatigue” [22]. Evidence from other disease areas suggests that PROMs are most effective when embedded within existing workflows and linked to specific clinical actions [18,23].

Second, patient-reported data may introduce interpretation challenges [39]. PROM scores are influenced by factors beyond AF itself, including comorbidities, psychological state, socioeconomic circumstances, and health literacy. Without appropriate contextualization and clinician training, PROM-triggered clinical actions could lead to unnecessary investigations or inappropriate treatment modifications.

Third, there is a risk that PROM integration may inadvertently widen health inequalities. Patients with lower digital literacy, older age, cognitive impairment, or limited language proficiency may be less able to complete PROMs, particularly when administered electronically [17,32,40]. Strategies to mitigate this—including paper-based alternatives, interviewer-administered PROMs, and simplified instruments—would need to be considered.

Fourth, the economic costs of PROM implementation are non-trivial. These include investment in digital infrastructure, electronic health record integration, staff training, and ongoing data management. In resource-constrained healthcare systems, these costs may not be immediately offset by improvements in clinical outcomes [29,32].

Fifth, unintended behavioral consequences should be anticipated. If PROM scores are linked to performance benchmarks or reimbursement models, there is a risk that clinicians may selectively administer PROMs to lower-risk patients or interpret results in ways that optimize metrics rather than patient care.

These considerations underscore the need for phased implementation, starting with pilot programs in selected clinical settings, accompanied by process evaluation to identify and mitigate unintended consequences before system-wide adoption.

### 4.3. Cross-Cultural and Health System Considerations

The applicability of the proposed framework may vary across healthcare systems with different levels of resources, infrastructure, and organization of care. The framework is most directly applicable to healthcare systems with established digital infrastructure and experience in collecting and integrating patient-reported data.

In resource-limited settings, barriers such as limited access to electronic health records, insufficient workforce capacity, and lack of standardized PROM implementation may affect feasibility. In addition, cultural differences in symptom perception, health reporting, and patient engagement may influence the interpretation and utility of PROM data.

Therefore, adaptation of the framework to local healthcare contexts may be required. Future implementation efforts should consider system-level characteristics, including digital maturity, reimbursement structures, and organizational workflows, as well as cultural factors that may affect patient-reported outcomes [32,41,42].

### 4.4. Future Research Directions

The proposed framework generates several testable research questions that should be addressed in future empirical studies:

1. Does routine PROM integration into AF clinical pathways improve patient-reported quality of life? A stepped-wedge cluster randomized trial comparing AF-CARE with versus without integrated PROMs, with the primary outcome of AFEQT overall score at 12 months and secondary outcomes of AF-related hospitalization rate and patient satisfaction, would provide the strongest evidence.
2. Does PROM-triggered clinical reassessment reduce unplanned hospitalizations? A pragmatic pre-post implementation study within a defined AF cohort, measuring hospitalization rates before and after the introduction of PROM-triggered reassessment protocols (e.g., clinical review triggered by ≥5-point AFEQT decline), could assess clinical impact with lower resource requirements.
3. What is the cost-effectiveness of PROM integration in AF care? A health economic evaluation alongside either of the above study designs, incorporating direct healthcare costs, PROM implementation costs, and quality-adjusted life years (QALYs), would provide the evidence base needed to inform reimbursement and policy decisions.
4. Is the framework acceptable and feasible across different healthcare systems? A multi-country qualitative study using semi-structured interviews with clinicians, policymakers, and patients across diverse healthcare settings (e.g., Latvia, Ireland, and Germany) would assess contextual barriers and facilitators to implementation.

A companion study is currently in preparation, describing the development and usability evaluation of an interactive dashboard that operationalizes the proposed framework [38,43,44]. This represents an initial step toward validation, with clinical implementation and outcome evaluation to follow.

## 5. Conclusions

Atrial fibrillation care appears to be at a transitional stage. The shift from volume-based to value-based healthcare requires reconsideration of how outcomes are defined and used in practice.

The present study suggests that currently available QoL instruments do not fully capture the multidimensional burden experienced by patients with AF. While widely used tools such as AFEQT provide important disease-specific insights, several domains—including cognition, sleep quality, sexual health, and economic burden—remain insufficiently represented.

The proposed framework may serve as a conceptual foundation for integrating PROMs into AF-CARE pathways, supporting more structured and patient-centered approaches. However, further empirical validation is required to assess its feasibility, effectiveness, and impact on clinical and health system outcomes. Within this approach, PROMs are viewed not only as measurement tools, but also as potential inputs for clinical decision-making, longitudinal monitoring, and resource planning.

This perspective is broadly consistent with contemporary guideline recommendations and value-based healthcare principles. However, its practical implications depend on how PROMs are implemented. Moving from measurement to meaningful use requires integration into clinical workflows, interpretation in context, and clear links to clinical action.

In practice, the value of PROMs will depend on whether they are used consistently and at clinically relevant time points. Indicators such as PROM completion rates, changes in AFEQT scores, and trends in AF-related hospitalizations may provide a starting point for evaluating implementation.

Overall, the challenge is not whether QoL should be measured, but how it can be incorporated into decision-making in a way that is both clinically meaningful and feasible in routine care. If we do not measure what matters to patients, we risk optimizing outcomes that patients themselves do not value.

## Figures and Tables

**Table 1:** Structural Characteristics and Domain Coverage of PROM Instruments in Atrial Fibrillation.

QoL Domain	AFEQT	EQ-5D	SF-36	MLHFQ	AFSS	Clinical Relevance	Policy Relevance
Physical functioning	Full	Full	Full	Full	Partial	Functional limitation	Rehabilitation planning
Symptoms	Full	Partial	Partial	Partial	Full	Core AF burden	Treatment evaluation
Emotional well-being	Full	Full	Full	Full	Partial	Mental health impact	Integrated care
Social functioning	Full	Partial	Full	Partial	Partial	Participation restriction	Social support systems
Cognitive function	Partial	None	Partial	Partial	None	Risk of decline	Screening strategies
Treatment satisfaction	Full	None	None	Partial	None	Adherence	Care quality indicators
Economic burden	Partial	None	None	None	None	Cost awareness	Health financing
Health perception	Full	Full	Full	Full	Partial	Overall status	Outcome monitoring
Sexual health	None	None	None	None	None	QoL determinant	Patient-centered care
Sleep quality	Partial	None	Partial	Partial	None	AF trigger	Preventive strategies

“Full” coverage was defined as comprehensive and validated assessment of a domain within the instrument; “partial” coverage referred to limited or indirect assessment; “none” indicated that the domain was not addressed.

**Table 2:** Clinical Relevance of PROM Domains in Atrial Fibrillation and Link to AF-CARE Framework.

PROM Domain	Clinical Relevance	Clinical Implication	AF-CARE Link
Symptoms	Reflects AF burden and symptom variability	Guides rhythm vs rate control decisions	R —Reduce Symptoms by rate and rhythm control
Physical functioning	Indicates functional limitation and disease impact	Supports treatment intensity adjustment	E—Evaluation and reassessment
Emotional well-being	Associated with psychological burden and symptom perception	May influence adherence and follow-up strategy	E—Evaluation and reassessment
Cognitive function	May indicate neurocognitive impairment related to AF	Triggers further neurological evaluation	C—Comorbidity and risk factors
Sleep quality	Linked to AF triggers and recurrence (e.g., sleep apnea)	Supports screening for comorbid conditions	C—Comorbidity and risk factors
Treatment satisfaction	Reflects perceived treatment effectiveness	May guide therapy optimization	E—Evaluation and reassessment
Economic burden	Indicates indirect disease impact and healthcare utilization	Relevant for system-level planning and resource allocation	E—Evaluation and reassessment
Health perception	Integrates overall patient-reported health status	Supports global disease severity assessment	E—Evaluation and reassessment

**Table 3:** Key Gaps in PROM-Based Assessment of Atrial Fibrillation and Their Clinical Implications.

Domain	Captured in Current PROMs	Diagnostic Consequence
Cognitive function	Partially	Underrecognition of cognitive decline and neurovascular risk
Sleep quality	Limited	Missed identification of AF triggers (e.g., sleep apnea)
Sexual health	Not captured	Incomplete assessment of quality of life and treatment impact
Economic burden	Limited	Underestimation of healthcare utilization and patient burden
Social functioning	Partially	Reduced understanding of patient participation and daily limitations

**Table 4:** Multilevel PROM Implementation Framework for Atrial Fibrillation Management.

Level	Implementation Actions	Measurable Indicators	AF-CARE Alignment
Clinical	Routine PROM collection at baseline and every 3–6 months; PROM-triggered clinical reassessment	PROM completion rates; AFEQT score changes ≥5 points; symptom-driven treatment adjustments	R—Rate/rhythm control; E—Evaluation and follow-up
Organizational	EHR-embedded PROMs; multidisciplinary interpretation; care coordination across settings	EHR integration rate; interdisciplinary referral frequency; care pathway adherence	C—Comorbidity management; A—Avoid stroke
Policy	Link PROMs to reimbursement models, national registries, and performance benchmarks	Reduction in AF-related hospitalizations; cost-effectiveness ratios; registry coverage	E—Evaluation; system-level VBHC alignment

AF-CARE: C = Comorbidity and risk factors; A = Avoid stroke; R = Rate and rhythm control; E = Evaluation and follow-up. EHR: Electronic Health Record; VBHC: Value-Based Healthcare.

## Data Availability

The original contributions presented in this study are included in the article. Further inquiries can be directed to the corresponding author.

## Supplementary Materials

The following supporting information can be downloaded at: https://www.mdpi.com/article/10.3390/diagnostics16091398/s1, Table S1: Comperative Psychometric Properties and Scoring Criteria of QoL Intruments Used in Atrial Fibrillation Populations.

## Abbreviations

The following abbreviations are used in this manuscript:

AF	Atrial Fibrillation
AF-CARE	Comorbidity, Avoid stroke, Rate/rhythm control, Evaluation
AFEQT	Atrial Fibrillation Effect on Quality of Life
AFSS	Atrial Fibrillation Severity Scale
EACTS	European Association for Cardio-Thoracic Surgery
ECG	Electrocardiogram
EHR	Electronic Health Record
ePROM	Electronic Patient-Reported Outcome Measure
EQ-5D	EuroQoL 5-Dimensions
ESC	European Society of Cardiology
ICHOM	International Consortium for Health Outcomes Measurement
MeSH	Medical Subject Headings
MLHFQ	Minnesota Living with Heart Failure Questionnaire
MOS	Medical Outcomes Study
OECD	Organisation for Economic Co-operation and Development
PROs	Patient-Reported Outcomes
PROMs	Patient-Reported Outcome Measures
QoL	Quality of Life
SF-36	Short Form Health Survey 36
VBHC	Value-Based Healthcare
WHO	World Health Organization
