# Supplementary material for: Integrating Patient-Reported Outcomes into Clinical Pathways in Atrial Fibrillation: A Framework Aligned with the AF-CARE Model
Source: Diagnostics (Basel). 2026 May 6;16(9):1398. doi: 10.3390/diagnostics16091398 (PMC13163613; doi:10.3390/diagnostics16091398)
Supplement: Supplementary file 1 [file diagnostics-16-01398-s001.zip › diagnostics-4263195-supplementary.pdf]

Appendix A. Supplementary Materials

Table S1. Comparative Psychometric Properties and Scoring Criteria of QoL Instruments Used in Atrial Fibrillation Populations.

Instrument	Domains/Subscales	Scoring Range	Psychometric Validation	Validation Population	MCID
AFEQT	Symptoms (4 items); Daily activities (8 items); Treatment concern (6 items); Treatment satisfaction (2 items)	0-100 (higher = better QoL)	Cronbach's alpha 0.88-0.95; test-retest reliability ICC 0.82-0.93 [9]	AF-specific; validated in diverse AF cohorts including paroxysmal, persistent, and permanent AF	5 points [12]
EQ-5D	Mobility; Self-care; Usual activities; Pain/discomfort; Anxiety/depression; VAS	Index: -0.59 to 1.0; VAS: 0-100 (higher = better)	Extensively validated; strong convergent validity; responsive to change in cardiac populations [10]	Generic; validated across multiple disease populations including cardiovascular conditions	0.05-0.08 (index); 7-12 (VAS)
SF-36	Physical functioning; Role-physical; Bodily pain; General health; Vitality; Social functioning; Role-emotional; Mental health	0-100 per subscale (higher = better); PCS/MCS summary scores (mean 50, SD 10)	Cronbach's alpha 0.78-0.93; well-established construct validity; high responsiveness in cardiac populations [11]	Generic; validated in >4,000 publications across clinical populations; used in AF trials as secondary endpoint	3-5 points per subscale
MLHFQ	Physical dimension (8 items); Emotional dimension (5 items); Overall score (21 items)	0-105 (lower = better QoL)	Cronbach's alpha 0.92; good discriminant validity between NYHA classes; moderate responsiveness in AF populations	Heart failure-specific; applied to AF populations with concurrent HF; limited AF-only validation	5 points
